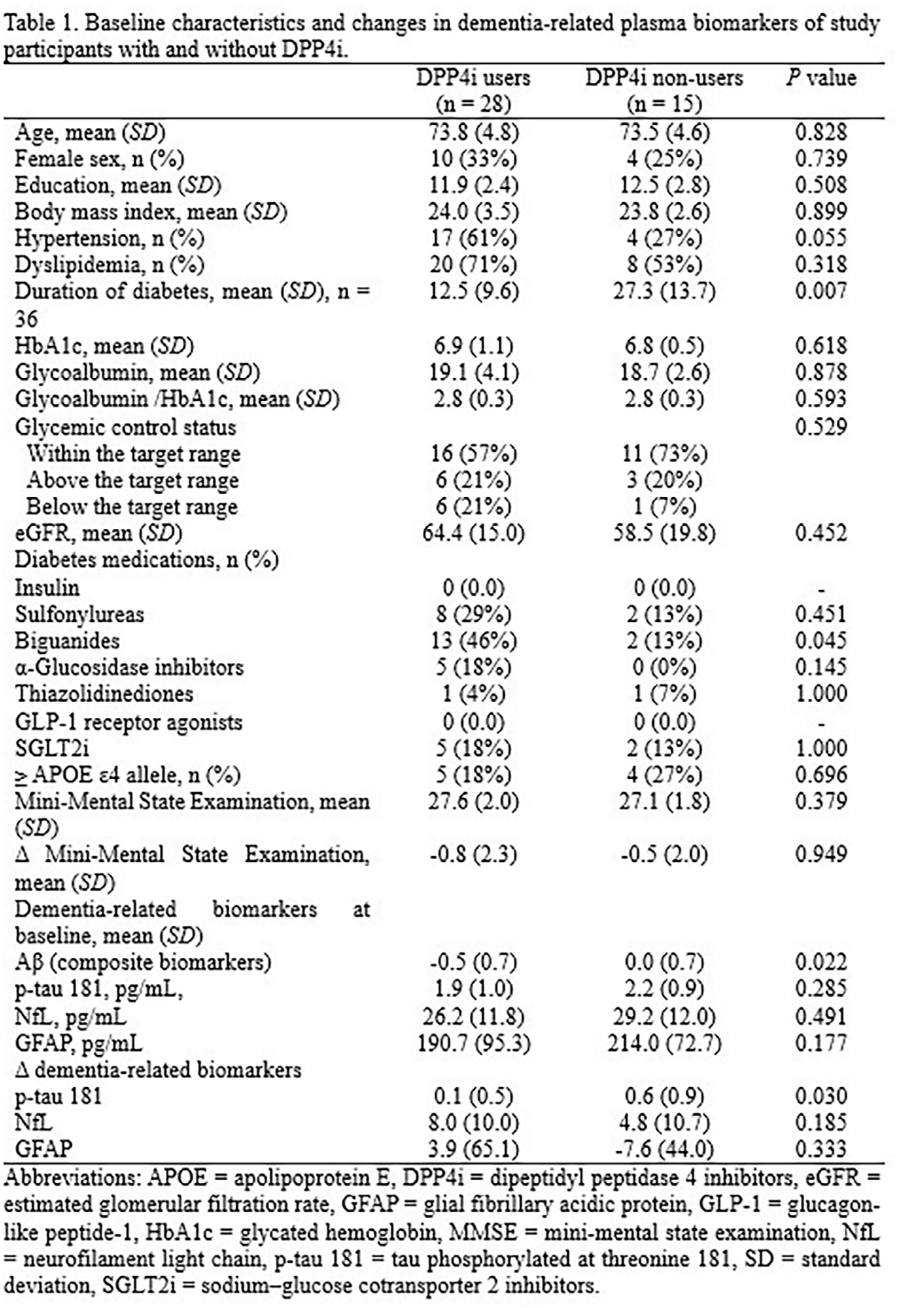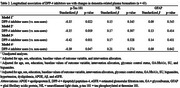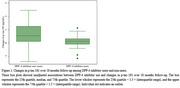# Association between DPP‐4 inhibitor use and changes in dementia‐related plasma biomarkers in older adults with type 2 diabetes: post‐hoc analyses of J‐MINT

**DOI:** 10.1002/alz70856_101474

**Published:** 2025-12-25

**Authors:** Taiki Sugimoto, Paul K Crane, Kazuaki Uchida, Kosuke Fujita, Yoko Yokoyama, Ayaka Onoyama, Yujiro Kuroda, Akinori Nakamura, Takuya Omura, Hisashi Noma, Elizabeth M Rhea, Hidenori Arai, Takashi Sakurai, J‐MINT study group

**Affiliations:** ^1^ University of Washington, Seattle, WA, USA; ^2^ National Center for Geriatrics and Gerontology, Obu, Aichi, Japan; ^3^ Institute of Statistical Mathematics, Tachikawa, Tokyo, Japan; ^4^ VA Puget Sound Health Care System, Seattle, WA, USA

## Abstract

**Background:**

Preclinical studies suggest that dipeptidyl peptidase‐4 (DPP‐4) inhibitors reduce brain β‐amyloid (Aβ) deposition, tau phosphorylation, and neuroinflammation. However, human data remain limited. This post‐hoc analysis of the Japan‐Multimodal Intervention Trial for Prevention of Dementia (J‐MINT) examined the association between DPP‐4 inhibitor use and changes in dementia‐related plasma biomarkers—phosphorylated tau 181 (*p*‐tau_181_), neurofilament light chain (NfL), and glial fibrillary acidic protein (GFAP)—in older adults with type 2 diabetes.

**Method:**

This study analyzed 43 participants with type 2 diabetes who completed plasma biomarker assessments at baseline and 18‐month follow‐up. Biomarkers (*p*‐tau_181_, NfL, GFAP) were measured using the single‐molecule array (Simoa™) platform. At baseline, the Aβ composite biomarker—calculated from APP669–711/Aβ_1–42_ and Aβ_1–40_/Aβ_1–42_—was measured using an immunoprecipitation‐mass spectrometry assay at Shimadzu Techno Research. To examine associations between DPP‐4 inhibitor use and biomarker changes, multiple linear regression analyses were performed. Model 1 was unadjusted; Model 2 included demographic variables (age, sex, education, intervention allocation) and baseline values; Model 3 added terms for glycemic control, glycemic variability (glycoalbumin/HbA1c), and sulfonylurea and biguanide use; Model 4 further adjusted for hypertension, dyslipidemia, presence of ≥1 *APOE* ε4 allele, Aβ composite biomarker, and estimated glomerular filtration rate.

**Result:**

Among 43 participants, 28 were DPP‐4 inhibitor users. At baseline, users had lower Aβ composite biomarker levels than non‐users (‐0.5 ± 0.7 vs. 0.0 ± 0.7, *P* = 0.02, Table 1). Over 18 months, users showed smaller increases in *p*‐tau_181_ (0.1 ± 0.5 pg/mL vs 0.6 ± 0.9 pg/mL, *P* = 0.03, Figure 1). Multiple regression analyses revealed a significant negative association between DPP‐4 inhibitor use and *p*‐tau_181_ changes across all models (Model 1, β = ‐0.35; Model 2, β = ‐0.35; Model 3, β = ‐0.42; Model 4, β = ‐0.39; all *P* < 0.05). No significant associations were observed for NfL or GFAP.

**Conclusion:**

Our findings align with preclinical evidence that DPP‐4 inhibitors may attenuate Alzheimer's disease pathology progression in individuals with type 2 diabetes. Further studies with larger sample sizes are needed to confirm these results.